# A new species of *Poropoea* Foerster from Africa (Hymenoptera, Chalcidoidea, Trichogrammatidae)

**DOI:** 10.3897/zookeys.658.11501

**Published:** 2017-02-23

**Authors:** Stefania Laudonia, Gennaro Viggiani, Silvano Biondi

**Affiliations:** 1 Department of Agriculture (BIPAF), University of Naples Federico II. Via Università 100, Portici (Naples) 80055. Italy; 2 Via E. di Velo 137, I Vicenza 36100- Italy

**Keywords:** Attelabidae, club unsegmented, key, leaf-rolling weevils, *Paratomapoderus brachypterus*

## Abstract

A new species of *Poropoea* Foerster (Hymenoptera, Chalcidoidea, Trichogrammatidae) was obtained from eggs of attelabid beetles (Coleoptera: Attelabidae) associated with *Combretum* sp. (Myrtales, Combretaceae). This species is described from Ogooué-Ivindo province in north-eastern Gabon. The new species is easily distinguished from the known members of the genus *Poropoea* by the following combination of characters: female antennal club unsegmented, premarginal vein of the fore wing with a nodular premarginal vein and the stigma of the stigmal vein black, the wing lacks the RS1 track; front and hind legs more robust than the middle leg and with coxa and femur markedly enlarged, and ovipositor exserted to one-third of the gaster length. Morphological features of this new species are discussed and illustrated. A key to females of *Poropoea* species lacking the Rs1 track in the fore wing has been constructed and is presented here.

## Introduction

The trichogrammatid genus *Poropoea* Foerster, 1851 (Hymenoptera, Trichogrammatidae) currently contains 19 species (Noyes 2017), well-known as egg parasitoids of leaf rolling attelabid beetles (Coleoptera: Attelabidae), which are distributed in the Palaearctic, Oriental, and Nearctic regions (Girault 1911, Silvestri 1916, Nowicki 1936, Hirose 1963, Viggiani 1968, Subba Rao 1969, Lin 1994, Luo and Liao 1994, Lou et al. 1996, Kobayashi and Kato 2004, Fursov 2007, Narendran and Hayat 2007, Hayat and Poorani 2013). A *Poropoea* sp. associated with *Apoderus humeralis*, currently included in the genus *Cycnotrachelodes* (Olivier) (Coleopera, Attelabidae) (Reckhaus 1997) was reported from the Afrotropical realm, Madagascar sub-region (Zwick 2009). Recently, female specimens of a *Poropoea* sp. have been collected in Gabon from eggs of *Paratomapoderus brachypterus* (Voss) (Coleoptera, Attelabidae, Apoderinae, Hoplapoderini), in leaf rolls of a species of *Combretum* Loefl. belonging to the Combretaceae. Their taxonomic study showed that they represent an undescribed species, clearly differentiated from all other species of the genus *Poropoea*. This new species, the first record of a member of the genus for the West-Central African sub-region, is described herein, and a key to the females of *Poropoea* species lacking the Rs1 track in the fore wing is given.

## Materials and methods

From a *Combretum* sp., 67 leaf rolls of *Paratomapoderus brachypterus* were sampled from 15 to 25 June 2016 in Gabon, Ogooué-Ivindo, Ivindo National Parc, Ipassa Makokou Strict Nature Reserve, Station de Recherche de Ipassa, 500m a.s.l; 0°30'43"N, 12°48'12"E. The emerged parasitoids, two specimens, were initially preserved in 70% alcohol. These specimens were later dissected and mounted on slides using balsam-phenol as permanent mounting medium. For the terminology, Doutt and Viggiani (1968) and Pinto (2006) were followed.

## Taxonomy

### Poropoea africana

Taxon classificationAnimaliaHymenopteraTrichogrammatidae

Laudonia & Viggiani sp. n.

http://zoobank.org/E2FA26B9-B944-4572-ABA3-902EAF14C926

Figs 1–6

#### Holotype

♀ (on slide). Gabon: Ogooué-Ivindo, Parc N. Ivindo, Station de Recherche de Ipassa, m a.s.l 500; 0°30'43"N, 12°48'12"E (DMS), June 2016, leg. Silvano Biondi. **Paratype**: 1 ♀, same data as holotype. Holotype and paratype will be deposited in the Entomological collection of the Dipartimento di Agraria dell'Università degli Studi “Federico II”, Portici, Napoli, Italia.

#### Diagnosis.

The new species is easily distinguished from the known members of the genus *Poropoea* by the following combination of characters: female antennal club unsegmented; premarginal vein of the fore wing with a basal “knot” and stigma of the stigmal vein black; lack of RS1 track in the fore wing; front and hind legs more robust than the middle ones, and with coxa and femur markedly enlarged; and ovipositor exserted for one-third of the gaster length. For the lack of the RS1 track on fore wing, *Poropoea africana* shares this character only with *Poropoea bella* Hayat et Poorani, *Poropoea longicornis* Viggiani, and *Poropoea orientalis* Subba Rao, but it is unique in having the club unsegmented, a nodular premarginal vein, and the coxa and femur of the front and hind legs markedly enlarged.

#### Description.

**Figure 1–6.**
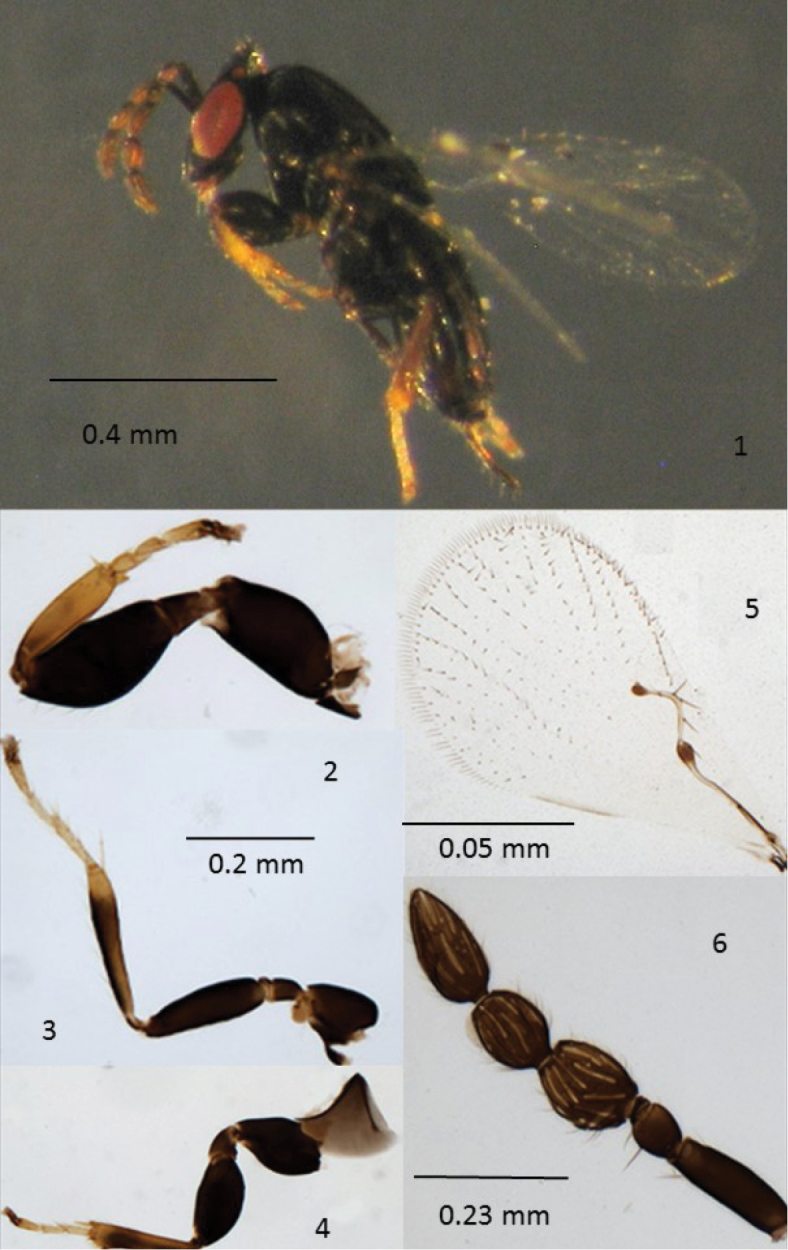
*Poropoea africana*, holotype female**. 1** adult **2** fore leg **3** middle leg **4** hind leg **5** fore wing **6** antenna.

**Female** (Fig. 1): Body length 1.18 mm. Body dark brown and shiny, ocelli and eyes red; antennae, legs (except the yellow-ochraceous tarsi, front tibia, distal part of middle and hind legs), and exserted part of the ovipositor, concolourous with the body. Fore wing hyaline but with basal half of the sub costal vein, base of premarginal vein and stigma of the stigmal vein dark brown. *Head* lenticular, 2.6 wider than long. Maxillary palps 2-segmented and labial palps vestigial. Mandibles with two external acute teeth. Antennal formula 1,1,(2), 2,1 (Fig. 6); scape narrow, three times as long as wide; pedicel small, almost as long as wide; two small anelli present; first funicular segment clearly wider than the second one and 1.3 as long as wide; second funicular segment smaller than the first, 1.2 as long as wide; club coniform, unsegmented, 1.6 as long as the second funicular segment; antennal segments with short and scanty setae; funicular segments with two and club with three rows of longitudinal sensilla. *Mesosoma* rather flat, same plane and slightly longer than metasoma; pronotum short, mid lobe of mesoscutum as long as scutellum but slightly longer and wider, both with a shallow reticulate sculpture and with two pairs of setae; mid lobe of mesoscutum and scutellum without a longitudinal median groove; metanotum very short, with 2-3 transversal thickenings; propodeum medially slightly longer than metanotum with a pair of large, ovoid spiracles near the anterior margin of the sclerite and a thickened strand starting from the internal spiracle margin and reaching the middle of the propodeum. Fore wing (Fig. 5), twice as long as broad, with subcostal, premarginal, marginal, and stigmal vein ratios 23:10:8:8, premarginal vein with a basal nodule and with one seta; arched marginal vein which fails to attain the anterior wing margin, with three setae, stigmal vein with a short seta in the middle; blade without cilia from base to below the level of stigmal vein, with 15 rows of cilia distinct reaching the distal margin of the wing and with few small cilia between them, vein track RS1 lacking; fringe very short, the longest cilia 12.5 times shorter than discal area width. Hind wing with two rows of cilia along anterior margin and two rows of cilia along the posterior margin. Fore leg (Fig. 2) evidently enlarged, with coxa and femur each 1.8 times as long as broad; femur with an outer margin convex; tibia as long as femur length, with its distal end provided with a single spine and a short and bifid apical spur; tarsus shorter than tibia (30:40) with first two tarsomeres sub-equal, approximately twice as long as wide, third tarsomere slightly longer. Middle leg (Fig. 3) thinner than the fore leg, with femur 3.6 times as long as wide; tibial spur a little shorter than basitarsus, the latter narrow and a little shorter than the following two segments combined. Hind leg (Fig. 4) with femur enlarged, twice as long as broad; tibia 1.2 times the femur length and distally provided with a whorl of short, coniform spines, and with a robust spur, shorter than basitarsus.

*Metasoma* shorter than mesosoma (48:41); ovipositor inserted at level of the first segment of gaster with the exserted part one-third as long as metasoma length; third valvulae nearly one-third the total length of the ovipositor; stylets very long.

**Male.** unknown.

#### Etymology.

The name refers to the continent of the collection site.

#### Distribution.

Gabon, West-Central Africa.

#### Remarks.

The new species is easily distinguished from the known members of the genus *Poropoea* The markedly enlarged coxa and femur of the front and hind legs is an interesting character which appears to be clear adaptations to host parasitisation [probably for body stability and propulsive efficiency of the ovipositor], and not reported for any other species of the genus *Poropoea* Similar features of the legs are present in the male of *Blastophaga psenes* (L.) (Hymenoptera, Chalcidoidea, Agaonidae), a well-known gall maker and pollinator of *Ficus carica* and in other fig-wasps only (Grandi 1929, Hill 1967).

Some characters of *Poropoea africana*, such as the antennal formula, the nodular premarginal vein, and the and modified legs, may suggest the inclusion of this species at least in a new subgenus, but this proposal is deferred until more material, including males, could be collected for a proper evaluation of these characters.

##### Key to females of *Poropoea* species lacking of the Rs1 track in the fore wing

**Table:** 

1	Antennal formula 1(scape), 1(pedicel), (2)(anelli), 3 (funicle), 2 (club)	**2**
–	Antennal formula 1, 1, (2), 2, (3) or 1, 1, (2), 2, 1	**3**
2	Exserted part of the ovipositor about as long as meso-and metathorax combined; funicular segments at least 2.5 times as long as wide	***Poropoea longicornis* Viggiani**
–	Exserted part of the ovipositor as long as head, meso-and metathorax combined, funicular segments a little more than 2 times as long as wide	***Poropoea orientalis* Subba Rao**
3	Antennula formula 1,1,(2), 2, (3); mid lobe of mesoscutum and scutellum with a longitudinal, median groove; normal legs	***Poropoea bella* Hayat & Poorani**
–	Antennal formula 1,1,(2), 2,1; mid lobe of mesoscutum and scutellum without a longitudinal, median groove; middle legs markedly smaller than fore and hind legs	***Poropoea africana* sp. n.**

## Supplementary Material

XML Treatment for Poropoea africana
